# Varicella zoster virus-associated morbidity and mortality in Africa – a systematic review

**DOI:** 10.1186/s12879-017-2815-9

**Published:** 2017-11-14

**Authors:** Hannah Hussey, Leila Abdullahi, Jamie Collins, Rudzani Muloiwa, Gregory Hussey, Benjamin Kagina

**Affiliations:** 1 Institute of Tropical Medicine and International Health, Charité-Universitätsmedizin Berlin, Berlin, Germany; 2 Vaccines for Africa Initiative, Division of Medical Microbiology & Institute of Infectious Disease and Molecular Medicine, University of Cape Town, Cape Town, South Africa; 3 Department of Biostatistics, School of Public Health, Harvard Medical School, Boston, MA USA; 4 Department of Paediatrics & Child Health, Groote Schuur Hospital, University of Cape Town, Cape Town, South Africa; 5 Vaccines for Africa Initiative, School of Public Health & Family Medicine, University of Cape Town, Cape Town, South Africa

**Keywords:** Varicella, Zoster, Shingles, Chickenpox, Africa, Epidemiology

## Abstract

**Background:**

Varicella zoster virus (VZV) causes varicella and herpes zoster. These vaccine preventable diseases are common globally. Most available data on VZV epidemiology are from industrialised temperate countries and cannot be used to guide decisions on the immunization policy against VZV in Africa. This systematic review aims to review the published data on VZV morbidity and mortality in Africa.

**Methods:**

All published studies conducted in Africa from 1974 to 2015 were eligible. Eligible studies must have reported any VZV epidemiological measure (incidence, prevalence, hospitalization rate and mortality rate). For inclusion in the review, studies must have used a defined VZV case definition, be it clinical or laboratory-based.

**Results:**

Twenty articles from 13 African countries were included in the review. Most included studies were cross-sectional, conducted on hospitalized patients, and half of the studies used varying serological methods for diagnosis. VZV seroprevalence was very high among adults. Limited data on VZV seroprevalence in children showed very low seropositivity to anti-VZV antibodies. Co-morbidity with VZV was common.

**Conclusion:**

There is lack of quality data that could be used to develop VZV control programmes, including vaccination, in Africa.

**Trial registration:**

PROSPERO 2015: CRD42015026144.

**Electronic supplementary material:**

The online version of this article (10.1186/s12879-017-2815-9) contains supplementary material, which is available to authorized users.

## Background

Varicella zoster virus (VZV), a member of the herpesviridiae family, is responsible for two distinct disease entities: varicella (chicken pox) and herpes zoster (shingles). VZV is a ubiquitous virus and both diseases of varicella and herpes zoster occur commonly throughout the world [1, 2]. Primary infection with VZV results in varicella, a usually benign disease of childhood characterized by fever and a pruritic vesicular rash. Complications, such as bacterial skin infections, pneumonia and encephalitis, can however, occur and may result in significant morbidity and mortality, particularly if the primary infection occurs in adults or those who are immunosuppressed [1].

Following the primary infection, VZV remains dormant in the host. With decreased immunity, as seen in elderly persons or with human immunodeficiency virus (HIV)-associated immunosuppression, VZV reactivates causing herpes zoster, a painful vesicular rash with a dermatomal distribution. A common complication of herpes zoster is post-herpetic neuralgia, which can result in significant morbidity. Eye involvement, including uveitis with the risk of blindness, can also occur. Occasionally herpes zoster may present without a rash (zoster sine herpete) and VZV neurological complications can sometimes occur in the absence of a rash [3]. The epidemiology of these atypical presentations of VZV is, however, still not clear. In Africa, risk factors for VZV include the rapidly growing elderly population [4] and a high HIV prevalence [5].

Molecular epidemiological analysis of VZV shows some evidence of geographical segregation, which may partly be explained by climatic conditions [6]. The VZV genotype M1, belonging to clade 5, has been associated with African origin [7]. Understanding the VZV genotypes distribution is an important factor to consider when implementing vaccination programs against the diseases in any geographical setting.

Safe and effective vaccines against both varicella and herpes zoster exist [2]. The varicella vaccine has also been shown to be safe and immunogenic in immunosuppressed children, including those with HIV (provided the child is not severely immunosuppressed and the CD4+ T-cell count is >15%) [8, 9]. The World Health Organisation (WHO) recommends routine childhood vaccination against varicella in certain settings: where varicella is ‘an important public health and socioeconomic problem’; vaccination is affordable; and high vaccination coverage can be sustained [9]. Settings that meet these WHO recommendations are mainly in high income countries, where routine varicella vaccination is now widespread [8]. This is in contrast to Africa, where other public health priorities, suboptimal healthcare infrastructure, inability to reach high vaccination coverage rates and lack of epidemiological data all contribute to the vaccine against VZV being rarely used.

The vaccine (live attenuated VZV) used against herpes zoster is similar to that for varicella, but the herpes zoster vaccine has 14-fold more plaque-forming units of the attenuated virus per dose [2, 10]. As of 2014, the WHO had not issued recommendations on the routine vaccination against herpes zoster, due to limited evidence [2].

In Africa, there are several other vaccine preventable diseases (VPD’s) associated with a greater public health burden [11, 12], compared to VZV-associated diseases. Immunization priorities and strategies are largely driven by the public health burden and the severity of VPD’s. Routine vaccination against VZV is therefore, not considered a priority in Africa. Although VZV-associated morbidity and mortality rates are generally low in most settings, both varicella and herpes zoster can cause considerable strain on healthcare systems and society, in the absence of preventative measures [1].

For Africa to design effective strategies that can mitigate the VZV-associated disease burden, it is crucial to understand the epidemiology of both varicella and herpes zoster on the continent. Most of the available and published VZV epidemiology data come from settings such as Europe and North America [8]. This epidemiological data on VZV in developed countries cannot be extrapolated to Africa because of several differences between the two settings, including climate, the HIV epidemic and malnutrition [13, 14]. In addition, suboptimal access to healthcare services may exacerbate the VZV-associated disease burden in Africa. Data from Brazil shows that varicella in the pre-vaccine period was associated with significant morbidity and mortality [15]. Similar findings are, therefore, expected from some countries in the African continent, such as South Africa.

Prior to this review, there has been no synthesised literature on VZV epidemiology in Africa. Our review aimed to address this gap in knowledge by describing the epidemiology of VZV in Africa, taking into account both varicella and herpes zoster. Because HIV/AIDS is prevalent in Africa, we were also interested in assessing the impact of the HIV/AIDS epidemic on the epidemiology of varicella and herpes zoster.

## Methods

### Protocol registration and publication

We registered and published a detailed description of the methods used for this systematic review [16].

### Study eligibility criteria

Studies were eligible for inclusion in this review if they reported the epidemiology of VZV in any age group, were conducted in any African country and reported any outcome of interest as defined in our protocol [16]. An additional criterion for inclusion was having a case definition for the diseases.

For varicella, included studies must have stated a case definition; failure to do which would result in exclusion. The case definition considered for varicella is the one used by the Centres for Disease Control and Prevention (CDC) is [17]:

- Clinical description: An acute illness with diffuse maculopapulovesicular rash, without other apparent cause.
- Laboratory criteria: Isolation of varicella virus from a clinical specimen, or varicella antigen detected by direct fluorescent antibody test, or varicella-specific nucleic acid detected by polymerase chain reaction (PCR), or significant rise in serum anti-varicella IgG antibody level by any standard serologic assay.

For herpes zoster, included studies must have stated the clinical signs (painful maculopapulovesicular rash, usually confined to a dermatome), as it has been shown to be pathognomonic [18].

### Systematic review outcomes

#### Primary outcomes

- Incidence or prevalence of varicella or herpes zoster
- Proportion of varicella or herpes zoster cases requiring hospitalization
- Mortality associated with varicella or herpes zoster

#### Secondary outcomes

- Proportion of varicella or herpes zoster morbidity or mortality associated with HIV/AIDS

### Literature search

Details of the literature search strategy are provided in the protocol [16]. We searched from the following databases: PubMed, Scopus, Africa-wide, Embase, WHOLIS, PDQ-Evidence, CENTRAL, CINAHL and Web of Science.

### Study selection

Cross-sectional, cohort and intervention studies were eligible for inclusion. All studies published from January 1974 to September 2015 were included without language restrictions. For non-English articles, Google translator software was used to translate to English, to enable us conduct a preliminary screening based on titles or abstracts. Subsequently, if the record needed further scrutiny, we contacted a scientist who is a native speaker of the language that needed translation to English. For articles in French that appeared likely eligible for inclusion, we sought translation support from a native speaker within our network of collaborators.

### Screening of the selected studies

**Fig. 1:**
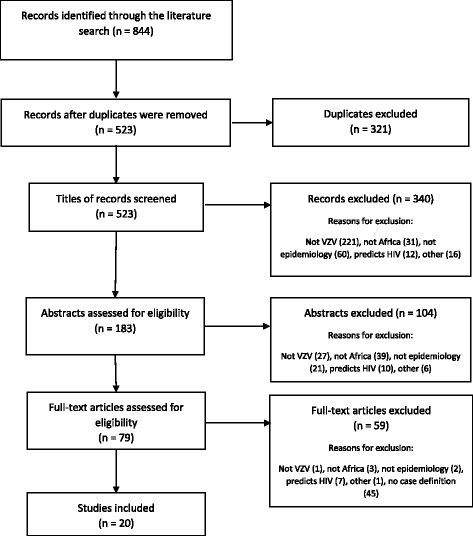
PRISMA flow diagram showing the study selection process. A total of 844 records were identified during the search procedures from the 9 databases. From the total records, 321 were duplicates and therefore excluded. The remaining 523 records were subjected to screening for inclusion/exclusion. Following the screening, 20 records met the inclusion criteria

The first (HSH) and the second (LHA) authors screened the literature search outputs using titles and abstracts. In addition, study setting, study design, methods as well as study outcomes were evaluated. The two authors then independently read through the full text of all potentially eligible studies to assess if inclusion criteria were met. Discrepancies between the two authors were resolved through discussion and consensus, with the assistance of the last author (BMK). The bibliographies of the included studies were screened for any other relevant studies. No additional studies were included in this review from the reference list of the selected studies. The Preferred Reporting Items for Systematic reviews and Meta-Analyses (PRISMA) flow diagram (Fig. 1) illustrates the process of the study screening and selection.

### Data collection

Data were independently extracted from the included studies by two reviewers, and recorded on a predesigned form. For more information on the data collection, see the protocol [16].

### Risk of bias assessment and strength of the cumulative results for included studies

We adapted the risk of bias and quality assessment tool developed by Hoy et al.*,* [19] and modified by others [20] for prevalence studies. Two authors (HSH and LHA) independently scored the risk of bias using the tool, and a κ agreement was calculated.

### Summary measures

The extracted data were heterogeneous and therefore, meta-analysis was not feasible.

### Synthesis and presentation of the results

Narrative reporting was used to describe the data from the studies. The findings of this systematic review are reported according to the PRISMA guidelines [21].

## Results

The complete raw data-set is available as a supplementary file (Additional file 1: Table S1).

### Study selection

The results of the searches from several database sources are summarised in a PRISMA flow diagram (Fig. 1). A total of 844 records were identified during the search procedures from the 9 databases. From the total records, 321 were duplicates and therefore excluded. The remaining 523 records were subjected to screening for inclusion/exclusion. Following the screening, 20 records met the inclusion criteria.

### Limited VZV epidemiology data in Africa

**Fig. 2:**
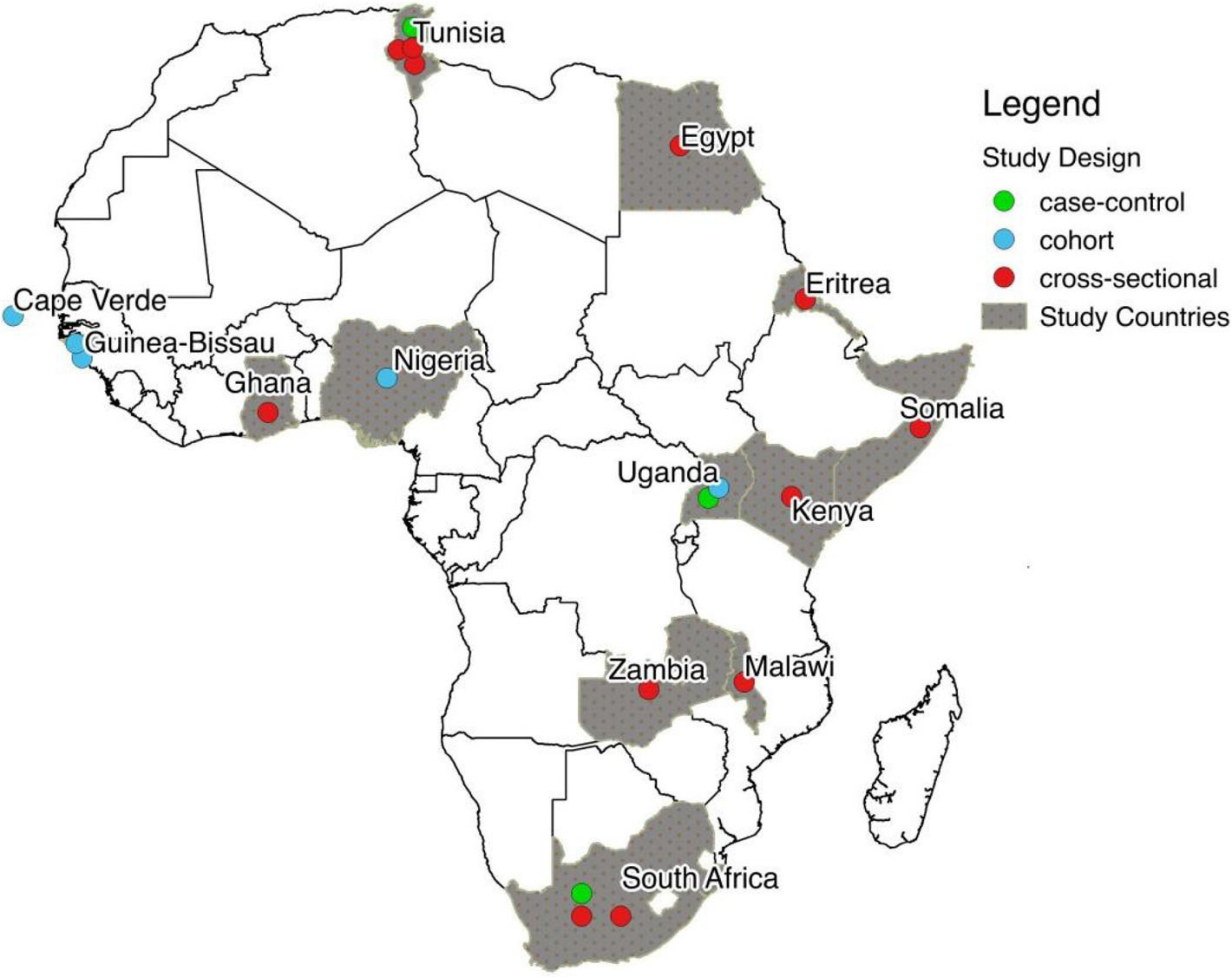
Map of Africa. Map of Africa showing the countries where included studies were conducted. The figure legend shows the types of study design. A total of 20 studies from 13 countries were included in the review. (Copyright was not required for this figure)

Our first aim was to establish which countries in Africa have reported published data on the VZV epidemiology between 1974 and 2015. There were 20 published studies conducted in 13 different African countries (24% of all countries on the continent) included in the final analysis (Fig. 2).

### Available VZV epidemiology data in Africa is mainly from cross-sectional studies

Study characteristics of the 20 included articles are described in Table 1. Twelve (60%) studies were conducted in health-care facilities, six (30%) studies were conducted in the community and the remaining two (10%) studies were conducted in both health-care facilities and communities simultaneously. Twelve (60%) studies were cross-sectional, five (25%) were cohort and three (15%) were case-control. Two studies were published in the 1980’s and one study was published in the 1990’s. The remaining seventeen studies (85%) were published between 2000 and 2015. The median sample size of participants in all studies was 305 (range 47–45,000).

**Table 1:** Study characteristics, including country and setting

Author (year of publication)	Citation number	Country	Community OR Health facility (setting)	Study Design	Sample size
Schoub, B. D., et al. (1985)	[37]	South Africa	Community	cross-sectional	244
Sixl, W. and B. Sixl-Voigt (1987)	[25]	Cape Verde	Health facility	cohort	380
Ghebrekidan, H., et al. (1999)	[45]	Eritrea	Community	cross-sectional	450
Poulsen, A., et al. (2002)	[22]	Guinea-Bissau	Community	cohort	37,400
Poulsen, A., et al. (2005)	[23]	Guinea-Bissau	Community	cohort	45,000
Selim, H. S., et al. (2007)	[35]	Egypt	Health facility	cross-sectional	322
Admani, B., et al. (2008)	[38]	Kenya	Health facility	cross-sectional	182
Compston, L. I., et al. (2009)	[28]	Ghana	Both	cross-sectional	412
Ajayi, G. O., et al. (2011)	[44]	Nigeria	Health facility	cohort	70
Hannachi, N., et al. (2011)	[42]	Tunisia	Health facility	cross-sectional	404
Ben Fredj, N., et al. (2012)	[33]	Tunisia	Both	case-control	102
Nahdi, I., et al. (2012)	[29]	Tunisia	Health facility	cross-sectional	126
Benjamin, L. A., et al. (2013)	[34]	Malawi	Health facility	cross-sectional	183
Nahdi, I., et al. (2013)	[30]	Tunisia	Health facility	cross-sectional	47
Schaftenaar, E., et al. (2014)	[41]	South Africa	Health facility	cross-sectional	405
Siddiqi, O. K., et al. (2014)	[32]	Zambia	Health facility	cross-sectional	331
Asiki, G., et al. (2015)	[39]	Uganda	Community	case-control	166
Laaks, D., et al. (2015)	[31]	South Africa	Health facility	case-control	129
Leung, J., et al. (2015)	[24]	Somalis living in Kenya	Community	cross-sectional	288
Rubaihayo, J., et al. (2015)	[26]	Uganda	Health facility	cohort	5972

From the 20 studies, a total population of 92,613 participants were studied. Two large surveillance studies from Guinea-Bissau [22, 23] contributed largely to the reported total population. 50% of the studies were based in urban areas (*n* = 10), 20% in rural areas (*n* = 4) while 10% in both rural and urban areas (*n* = 2). 10% of the studies were conducted in peri-urban areas. One study was partially conducted in an urban area and a refugee camp [24] while the specific information on the area of study was missing for one study [25].

### Burden of VZV-associated disease in Africa is high but variable

**Fig. 3:**
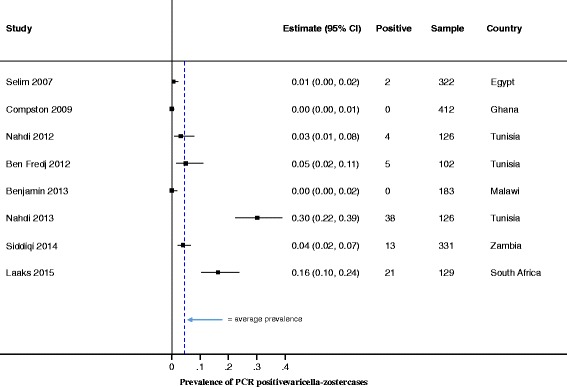
VZV Prevalence, as diagnosed by PCR. Forest plot of VZV prevalence, as diagnosed by PCR. Due to the heterogeneity of the prevalence data, we did not conduct meta-analyses and, therefore, do not report on the pooled prevalence

**Fig. 4:**
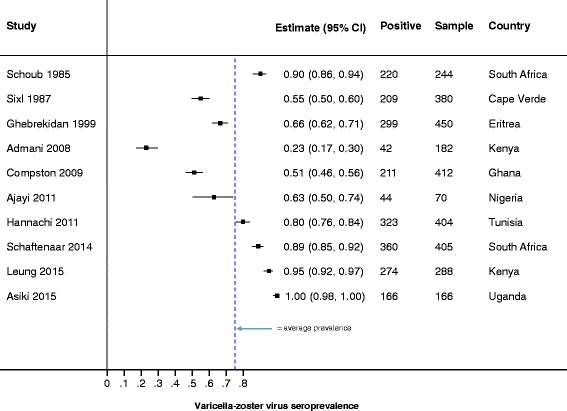
VZV Prevalence, as diagnosed by serology. Forest plot of VZV prevalence, as diagnosed by serology. Due to the heterogeneity of the prevalence data, we did not conduct meta-analyses and therefore, do not report on the pooled prevalence

We evaluated the reported incidence, prevalence, hospitalization and mortality data from the included studies (Table 2 and Figs. 3 and 4 **)**. Across the included studies, variable case definitions for VZV disease were used, and these can be broadly categorised into two types: serological and clinical. Only two (10%) studies combined both serological and clinical criteria for the VZV case definition. According to our results, the available data from the 13 (65%) studies on the burden of VZV disease in Africa is mostly from adult populations.

**Table 2:** Burden of VZV in Africa

Author (year of publication)	Study population	Case definition	Incidence (per 100,000)	Hospitalization & Mortality
Varicella incidence				
Poulsen, A., et al. (2002)	children & adults	clinical & serology	441 per 100,000	NS; 0%
Poulsen, A., et al. (2005)	children & adults	clinical & serology	3420 per 100,000	NS; 0.13%
Zoster incidence				
Rubaihayo, J., et al. (2015)	adults	clinical	751 per 100,000	NS; NS

#### Incidence

Of the 20 included studies, only 3 (15%) reported incidence of VZV. Two of the three incidence studies reported varicella in Guinea Bissau, and these studies were published by the same authors and used a combination of serology and clinical tests to diagnose VZV (Table 2 and Figs. 3 and 4 **)**. Using population registers to estimate the denominator, ncidences of 441 per 100,000 and 3420 per 100,000 were reported by Poulsen et al.*,* 2002 and 2005 respectively [22, 23]. As the denominator was estimated by the authors, the accuracy of the incidences reported is not clear. The median age for the varicella cases reported by the incidence studies were 3 [22] and 4.4 years [23]. Co-morbidity reported by the two incidence studies were pneumonia at 1.8% and 10% [22, 23] respectively. Other complications reported by Poulsen et al.*,* 2002 were bacterial skin infections (43.6%), cough (23.6%), conjunctivitis (4%) and diarrhoea (1.8%). Poulsen et al.*,* 2005 reported a case-fatality rate of 0.13%. Poulsen et al.*,* 2002 did not report mortality.

The study by Rubaihayo et al.*,* 2015 diagnosed herpes zoster clinically and reported an annual incidence of 751 per 100,000 in Ugandan HIV positive patients [26]. Median age of participants in this study was 32 years (range 26–39) and median CD4 at antiretroviral treatment (ART) initiation was 128 cells/mm^3^.

#### Prevalence

There were eight (40%) studies that reported prevalence of VZV-associated disease quantified by viral detection using PCR. Although expensive and requiring greater technical expertise and resources, PCR tests, whether quantitative or qualitative, show superior sensitivity and specificity compared to antibody detection tests [27]. Of the eight studies that used PCR, three were quantitative [28–30], two qualitative [31, 32], and the remaining three did not specify if the PCR used was quantitative or qualitative [33–35]. Four of the eight studies were performed on CSF (in patients that presented with signs of central nervous system [CNS] infections), whereas two studies were performed on ocular fluids (mostly from patients with uveitis). The eight studies found a VZV prevalence that ranged from 0% to 90% (Fig. 3). This big variation could be explained by the fact that these studies had recruited highly selected patient populations (i.e. patients presenting to healthcare facilities with a possible CNS infection or uveitis). It is unlikely that the VZV prevalence reported from these patients can, therefore, be extrapolated to the general population.

#### Seroprevalence

Evaluation of antibodies to VZV (IgG, IgM or both) was reported in ten studies (Fig. 4). IgG provides an indication of previous infection or vaccination, and therefore immunity, while IgM suggests recent or acute infection [36]. Nine of the ten studies measured IgG, and of these, two also tested IgM. The nine studies used the enzyme linked immunoassay for the antibody measurements, while the tenth study used the complement fixation method without specifying the type of antibody measured. A combination of commercially available kits and inhouse immunoassays were used and there was high variability in how the ten studies tested for anti-VZV antibodies (IgG or IgM). In addition, cut-offs for anti-VZV antibodies positivity were not provided except by Schoub et al.*,* 1985 who indicated that a 0.15 regression value of the EIA was the cut-off positivity value [37]. The assay variability and lack of defined positivity cut-offs partly contributed to our inability to perform a meta-analysis on the seroprevalence in this review. Seroprevalences ranged from 21.9% (in hospitalized children with co-morbidities in Kenya [38]) to 100% (elderly patients in rural Uganda [39]). In general, and as expected in settings without routine vaccination, children showed lower proportions of seropositivity to VZV than adults (Fig. 4).

### VZV burden and co-morbidities, including HIV

For these results, we focused on the studies that used a laboratory detection of the VZV in quantifying the burden (Table 3). Our limited data did not allow us to establish an association between VZV burden and the HIV prevalence at a population level (Table 3). One study, however, suggested that HIV infection was associated with VZV seropositivity [28]. Compston et al.*,* 2009 compared the VZV seroprevalence in healthy HIV uninfected blood donors (45%) with symptomatic HIV patients (57.2%), and found the odds ratio for having VZV antibodies was 1.6 (95% CI, 1.1–2.6) [28].

**Table 3:** HIV and other co-morbidities. VZV prevalence based on detection of the virus and HIV burden

Author (year of publication)	HIV positive participants (%)	VZV Prevalence (%)	Other co-morbidities
Selim, H. S., et al. (2007)	.	0.6	meningitis/encephalitis
Ben Fredj, N., et al. (2012)	0	4.9	multiple sclerosis
Nahdi, I., et al. (2012)	0	3.2	“acute neuromeningeal disorder”
Benjamin, L. A., et al. (2013)	68	0	viral meningitis
Nahdi, I., et al. (2013)	0	29.8	uveitis
Siddiqi, O. K., et al. (2014)	100	3.9	CNS infections
Laaks, D., et al. (2015)	45.8	0–32	uveitis

Nine (45%) of the 20 included studies evaluated VZV burden in HIV infected populations. As an example, Rubaihayo et al.*,* 2015 assessed the trends of opportunistic infections among HIV-infected persons in Uganda and found that the VZV incidence was 1340 per 100,000 at the start of the study in 2002 in the pre-ART era, compared to the incidence of 330 per 100,000 in 2013 in the ART era [26].

Only one study reported VZV seropositivity (IgG) data in pregnant women and found it to be 80.9% in Tunisia [40]. One study, conducted among hospitalized children in Kenya reported the rates of seroprotective antibodies against VZV as 21.9%, 24.1% and 25.0% amongst those: infected with HIV, with malignancies and with severe malnutrition, respectively [38]. In the four studies that looked at VZV causing CNS infections, the prevalences ranged from 0% (of viral meningitis in adults in Malawi [34]) to 3.9% (in Zambian adults with CNS infections caused by VZV [32]). Amongst those who presented with seizures, VZV was diagnosed in 23.1% [32]. The patients were highly immunosuppressed (median CD4 count was 89) and the case fatality rate for VZV CNS infections was 30.8% [32] [Siddiqi, personal communication, 13 October 2016]. In immunocompetent patients in Tunisia, 29.8% of uveitis cases were caused by VZV [30], while in South Africa, 32% of uveitis cases were caused by VZV [31].

Most co-morbidities diagnosed alongside varicella or herpes zoster required hospitalization (e.g. severe malnutrition, meningitis) (Table 4). This may partly explain why most included studies were conducted in health facilities.

**Table 4:** HIV and other co-morbidities

Author (year of publication)	HIV positive participants (%)	Other co-morbidities
Schoub, B. D., et al. (1985)	.	
Sixl, W. and B. Sixl-Voigt (1987)	.	febrile illness
Ghebrekidan, H., et al. (1999)	.	
Poulsen, A., et al. (2002)	.	
Poulsen, A., et al. (2005)	.	
Admani, B., et al. (2008)	64	severe malnutrition, malignancies
Compston, L. I., et al. (2009)	29	
Ajayi, G. O., et al. (2011)	100	
Hannachi, N., et al. (2011)	.	pregnancy
Schaftenaar, E., et al. (2014)	100	
Asiki, G., et al. (2015)	16	stroke
Leung, J., et al. (2015)	.	
Rubaihayo, J., et al. (2015)	100	

### Risk factors for VZV infection

Several studies commented on the poor patient history of previous VZV infection, possibly due to recall bias [41], and patient history often did not correspond with serology [37, 42].

Crowded living conditions fuel the spread of VZV, and thus affect VZV seroprevalence. VZV seropositive status was associated with a higher number of individuals per household in rural South Africa [41]. A study amongst Somali refugees in Kenya noted high (94–96%) VZV seroprevalence rates in crowded refugee camps, that had experienced varicella outbreaks [24, 43].

### Risk of bias and quality assessment

A risk of bias and quality assessment was conducted independently by two raters using a modified assessment tool adapted from Hoy et al. [19]. Thereafter, Cohen’s κ was run to determine the inter-rater agreement. Moderate agreement (κ =0.510) was found (Additional file 2: Table S2).

Using the average score of the two independent raters, 8 studies had a score of 6–8 points, suggesting that they were at a moderate risk of bias, while 12 studies had a score of 8–10 points, implying that they were a low risk of bias.

## Discussion

We successfully conducted a systematic review on the epidemiology of VZV in Africa. A high but variable burden of VZV-associated disease was found. In this review, we observed high VZV seroprevalence (IgG) in adults. This review identified limited VZV seroprevalence data in children which was insufficient to compare with data from adults. One study by Adamani et al. showed relatively low rates of detectable antibodies in children [38]. In contrast, high rates of detectable VZV antibodies in adults were reported [24, 25, 28, 37, 39, 41, 42, 44, 45]. Because vaccination against VZV is not routine in Africa, we presume the high seroprevalence rates in adults indicates previous natural exposure or infection.

Although this review identified limited data on VZV in children, increasing age, even in adults was found to be associated with increased VZV seropositivity. A South African study reported that only a moderately high proportion of young adults had detectable VZV antibodies, compared to all individuals at 60 years of age in the same setting [37]. The VZV seropositive status was also found to be independently associated with age in HIV positive patients [41]. Taken together the VZV seropositivity results reported in this review suggests that primary VZV infection occurs at a later age in Africa, compared to other regions [1]. Interestingly later acquisition of varicella is thought to be possibly protective against developing shingles, as immunity developed during the initial infection could last longer [46].

Climatic conditions are known to influence the transmission of VZV [8]. Africa has widely varying climates, including large areas of tropical and sub-tropical climates that may result in a lower transmission of VZV and therefore primary infection at older ages, as has been seen in studies from India and South East Asia [14, 47]. Our results corroborate those of Lee et al.*,* who reported VZV seroconversion in Asian tropical countries peaking in adolescence and adulthood [14]. In contrast, seroconversion with VZV appears to peak during childhood in temperate countries [48].

Exposure to VZV is cumulative over time and seroconversion, therefore, is expected to increase with age. Seropositivity depends on what antibody level cut offs were used, and were unfortunately not reported for most studies in this review. Understanding the age of primary VZV infection in a given setting is crucial in the design of an optimal vaccination strategy. The only two studies that looked at varicella incidence (both from Guinea-Bissau) reported high incidence rates in both children and adults [22, 23]. The incidence rates reported in Guinea-Bissau seem higher than those observed in some temperate climates for a similar age group, [49, 50]. However, the Guinea-Bissau incidence rates are comparable to pooled incidence rates reported in Latin America [51].

Climatic conditions vary greatly between and within African countries, and, therefore, the data from Guinea-Bissau cannot be extrapolated to the whole continent. In addition, high levels of household overcrowding might negate the effect of a tropical climate on VZV transmission [8]. There were, however, eight studies that tested for VZV using PCR, as well as two that utilised IgM, which gave some indication of recent or current infection. Of the eight studies that utilised PCR, all excluding the Compston et al., 2009 study, were all in patients suffering from neurological or ocular diseases, and, therefore, cannot be extrapolated to the general population.

Important risk factors in the development of herpes zoster include advanced age and altered cell mediated immunity, as seen in malignancy, immunosuppressive treatment and HIV infection [36]. HIV positive individuals are thought to have a 12–17-fold greater risk of developing zoster [13]. In Sub-Saharan Africa where the burden of HIV is high, zoster has a high positive predictive value for underlying HIV infection [13]. A study conducted in Rwanda after the 1994 genocide found that amongst HIV positive patients, post-traumatic stress disorder was significantly associated with zoster (the study used patient self-reports to diagnose zoster and was, therefore, not included in this review) [52]. In a study amongst HIV positive patients in Zambia with CNS infections, VZV was the fourth most common viral infection and VZV case fatality rates were high [32]. In comparison, a study looking at viral meningitis in Malawi, where more than half of the patients were HIV positive, detected no VZV co-infection [34]. This was unexpected as VZV is a common identifiable cause of viral meningitis in industrialised countries [34]. A study by Compston et al. found that HIV was associated with VZV seropositivity [28]. The authors hypothesized that in individuals, who were previously exposed to VZV but remained seronegative, HIV-driven immune dysfunction leads to relatively small increases in VZV replication and reactivation. This in turn results in boosting of VZV antibodies and higher VZV seroprevalences in HIV [28].

The widespread and increasing availability of antiretroviral treatment (ART) in Africa [5] will likely have a positive impact on VZV epidemiology. Restored immune function on ART is thought to be protective against herpes zoster [53]. This is observed in Rubaihayo et al.*,* study where the VZV incidence dramatically decreased from the pre-ART era, compared to the ART era [26]. These findings are consistent with data from HIV positive patients in the USA [54]. The development of zoster while on ART has been suggested to be a marker of non-adherence to ART [55]. Taken together, these results suggest that HIV treatment programs should be strengthened when establishing vaccination programs against VZV in African countries. It must also be noted that as ART is allowing HIV positive people to live longer, the HIV population in Africa is aging [56]. The implications of an ageing HIV positive population on the epidemiology of zoster are not clear.

Optimal vaccination programmes against VZV are effective in mitigating the disease burden [8]. There are currently no routine vaccination programs against VZV in any African county unlike many developed countries, that have adopted a universal vaccination policy against varicella [8]. In 2006, some developed countries reported a case fatality rate for varicella of 2–4 per 100,000 [50, 57]. Slightly higher case fatality rates of 11 per 100,000 were reported in Latin America where vaccination programs have been introduced in some countries [51]. However, as shown in our review, case fatality rates of up to 130 per 100,000 were reported in Guinea-Bissau [23]. The VZV associated mortality is known to be about 30 times higher in adulthood than childhood [1]. We were not able to find any reported rates of hospitalization from our included studies. Rates of complications, including the development of post-herpetic neuralgia, were also not reported.

Our review is limited by a paucity of data on VZV in Africa. Between 1974 and 2015, only 20 studies from 13 countries met the inclusion criteria for our review. The countries that contributed to the review may not be representative of the continent as a whole [58]. As our review included only published studies, and did not consider grey literature, there is potential for bias in our findings. In addition, the quality of the included studies is also poor, as the review mostly consisted of cross-sectional studies that depended on serology for their case definition. The serological tests were mainly enzyme linked assays detecting IgG, lacked information on positivity cut-offs and the assays do not discriminate current infection from previous infection, previous exposure or vaccination. Furthermore, many of the studies were conducted in a health facility on patients already hospitalized with complications of possible VZV. While this does give us some indication of the burden that VZV imposes on the health system, the prevalences and mortality rates obtained from these studies cannot be extrapolated to the general population.

## Conclusion

Effective vaccines for both varicella and herpes zoster exist. We observed low and high levels of VZV seropositvity in children and adults respectively. The observations are worrisome, particularly in the context of a high HIV burden and an ageing population in Africa, as it suggests some primary exposure to VZV may be happening in adulthood. Primary VZV infection is more severe in adults than children. The resultant increased risk of morbidity and mortality from VZV could be mitigated by vaccines. Where universal varicella vaccination may not be feasible, such as in most African countries, vaccination of high risk individuals, such as healthcare workers, immunocompromised patients and their household contacts has been suggested [8]. But improved surveillance of VZV-associated diseases and cost-effectiveness data on the continent is first needed before vaccination can be considered. Future research must focus on generating quality primary data and feasibility of interventions on VZV in Africa.

## Additional files

Additional file 1: Table S1. Varicella in Africa – raw dataset. Data extracted from the included studies. (XLSX 44 kb)

Additional file 2: Table S2. Bias Scoring Agreement. Interrater agreement of risk of bias and quality assessment. (DOCX 11 kb)

## Abbreviations

AIDS: Acquired immunodeficiency syndrome
ART: Antiretroviral treatment
CDC: Centres for Disease Control and Prevention
HIV: Human immunodeficiency virus
PRISMA: Preferred Reporting Items for Systematic reviews and Meta-Analyses
VZV: Varicella zoster virus
WHO: World Health Organisation

## References

[1] Heininger U, Seward JF (2006). Varicella. Lancet Lond Engl.

[2] World Health Organization. Varicella and herpes zoster vaccines: WHO position paper, 20 June 2014, vol. 89, 25 (pp. 265–288) [Internet]. WHO. [cited 12 Aug 2015]. Available from: http://www.who.int/wer/2014/wer8925/en/.

[3] Gilden DH, Dueland AN, Devlin ME, Mahalingam R, Cohrs R (1992). Varicella-zoster virus reactivation without rash. J Infect Dis.

[4] Velkoff VA, Kowal PR. Aging in sub-Saharan Africa: the changing demography of the region [Internet]. National Academies Press (US); 2006 [cited 6 Sep 2016]. Available from: http://www.ncbi.nlm.nih.gov/books/NBK20301/.

[5] Global AIDS Update 2016 | UNAIDS [Internet]. [cited 2016 Aug 19]. Available from: http://www.unaids.org/en/resources/documents/2016/Global-AIDS-update-2016

[6] Quinlivan M, Hawrami K, Barrett-Muir W, Aaby P, Arvin A, Chow VT (2002). The molecular epidemiology of varicella-zoster virus: evidence for geographic segregation. J Infect Dis.

[7] Schmidt-Chanasit J, Ölschläger S, Günther S, Jaeger G, Bleymehl K, Schäd SG (2008). Molecular analysis of Varicella-zoster virus strains circulating in Tanzania demonstrating the presence of genotype M1. J Clin Microbiol.

[8] SAGE Working Group on Varicella and Herpes Zoster Vaccines (2014). Background paper on Varicella vaccine [internet].

[9] World Health Organization. Weekly epidemiological record, Varicella vaccines - WHO position paper. 1998, 73, 241-248 [Internet]. 1998 [cited 8 Sep 2015]. Available from: http://www.who.int/immunization/wer7332varicella_Aug98_position_paper.pdf.

[10] Levin MJ, Oxman MN, Zhang JH, Johnson GR, Stanley H, Hayward AR (2008). Varicella-zoster virus-specific immune responses in elderly recipients of a herpes zoster vaccine. J Infect Dis.

[11] Miller MA, Sentz JT. Vaccine-preventable diseases. In: Jamison DT, Feachem RG, Makgoba MW, Bos ER, Baingana FK, Hofman KJ, et al., editors. Dis. Mortal. Sub-Sahar. Afr. [Internet]. 2nd ed. Washington (DC): World Bank; 2006 [cited 31 Aug 2017]. Available from: http://www.ncbi.nlm.nih.gov/books/NBK2284/. 21290645

[12] Arevshatiana L, Clementsb C, Lwangac S, Misored A, Ndumbee P, Sewardf J, et al. An evaluation of infant immunization in Africa: is a transformation in progress? [Internet]. World Health Organization; 2007 Jun. Report No.: Volume 85, Number 6. Available from: http://www.who.int/bulletin/volumes/85/6/06-031526/en/. 10.2471/BLT.06.031526 PMC2636339 17639242

[13] Thomas SL, Hall AJ (2004). What does epidemiology tell us about risk factors for herpes zoster?. Lancet Infect Dis.

[14] Lee BW (1998). Review of varicella zoster seroepidemiology in India and Southeast Asia. Trop. Med. Int. Health TM IH..

[15] de Martino MA, Carvalho-Costa FA (2016). Varicella zoster virus related deaths and hospitalizations before the introduction of universal vaccination with the tetraviral vaccine. J Pediatr.

[16] Hussey HS, Abdullahi LH, Collins JE, Muloiwa R, Hussey GD, Kagina BM. Varicella zoster virus-associated morbidity and mortality in Africa: a systematic review protocol. BMJ Open [Internet]. 2016;6 [cited 21 Aug 2016]. Available from: http://www.ncbi.nlm.nih.gov/pmc/articles/PMC4838733/. 10.1136/bmjopen-2015-010213 PMC4838733 27098823

[17] Varicella | 2010 Case Definition [Internet]. [cited 2015 Aug 17]. Available from: http://wwwn.cdc.gov/nndss/conditions/varicella/case-definition/2010/

[18] Centers for Disease Control and Prevention. Shingles (Herpes Zoster) Diagnosis & Testing [Internet]. Available from: http://www.cdc.gov/shingles/hcp/diagnosis-testing.html

[19] Hoy D, Brooks P, Woolf A, Blyth F, March L, Bain C (2012). Assessing risk of bias in prevalence studies: modification of an existing tool and evidence of interrater agreement. J Clin Epidemiol.

[20] Werfalli M, Musekiwa A, Engel ME, Ross I, Kengne AP, Levitt NS (2014). The prevalence of type 2 diabetes mellitus among older people in Africa: a systematic review study protocol. BMJ Open.

[21] PLOS Medicine: Preferred Reporting Items for Systematic Reviews and Meta-Analyses: The PRISMA Statement [Internet]. [cited 10 Oct 2016]. Available from: http://journals.plos.org/plosmedicine/article?id=10.1371/journal.pmed.1000097 10.1371/journal.pmed.1000097 PMC2707599 19621072

[22] Poulsen A, Qureshi K, Lisse I, Kofoed P-E, Nielsen J, Vestergaard BF (2002). A household study of chickenpox in Guinea-Bissau: intensity of exposure is a determinant of severity. J Inf Secur.

[23] Poulsen A, Cabral F, Nielsen J, Roth A, Lisse IM, Vestergaard BF, et al. Varicella zoster in Guinea-Bissau: intensity of exposure and severity of infection. Pediatr Infect Dis J. 2005; 10.1097/01.inf.0000151034.15747.4a 15702036

[24] Leung J, Lopez A, Mitchell T, Weinberg M, Lee D, Thieme M (2015). Seroprevalence of Varicella-zoster virus in five US-bound refugee populations. J Immigr Minor Health.

[25] Sixl W, Sixl-Voigt B (1987). Serological screenings of various infectious diseases on the Cape Verde Islands (West Africa). J Hyg Epidemiol Microbiol Immunol.

[26] Rubaihayo J, Tumwesigye N, Konde-Lule J. Trends in prevalence of selected opportunistic infections associated with HIV/AIDS in Uganda. BMC Infect Dis. 2015;15 10.1186/s12879-015-0927-7 PMC4408591 25879621

[27] Chickenpox | Interpreting Laboratory Tests | Varicella | CDC [Internet]. [cited 31 Aug 2017]. Available from: https://www.cdc.gov/chickenpox/hcp/lab-tests.html

[28] Compston LI, Li C, Sarkodie F, Owusu-Ofori S, Opare-Sem O, Allain J-P (2009). Prevalence of persistent and latent viruses in untreated patients infected with HIV-1 from Ghana. West Africa J Med Virol.

[29] Nahdi I, Boukoum H, Nabil Ben Salem A, Ben Romdane F, Hammami S, Chebel S (2012). Detection of herpes simplex virus (1 and 2), varicella-zoster virus, cytomegalovirus, human herpesvirus 6 and enterovirus in immunocompetent Tunisian patients with acute neuromeningeal disorder. J Med Virol.

[30] Nahdi I, Abdelwahed RB, Boukoum H, Bressollette-Bodin C, Attia S, Yahia SB (2013). Herpesvirus detection and cytokine levels (IL-10, IL-6, and IFN-γ) in ocular fluid from Tunisian immunocompetent patients with uveitis. J Med Virol.

[31] Laaks D, Smit DP, Harvey J. Polymerase chain reaction to search for herpes viruses in uveitic and healthy eyes: a south African perspective [internet]. Afr Health Sci. 2015; Available from: http://www.ajol.info/index.php/ahs/article/download/121827/111288 10.4314/ahs.v15i3.7 PMC4765463 26957961

[32] Siddiqi OK, Ghebremichael M, Dang X, Atadzhanov M, Kaonga P, Khoury MN (2014). Molecular diagnosis of central nervous system opportunistic infections in HIV-infected Zambian adults. Clin Infect Dis Off Publ Infect Dis Soc Am.

[33] Ben Fredj N, Rotola A, Nefzi F, Chebel S, Rizzo R, Caselli E (2012). Identification of human herpesviruses 1 to 8 in Tunisian multiple sclerosis patients and healthy blood donors. J Neuro-Oncol.

[34] Benjamin LA, Kelly M, Cohen D, Neuhann F, Galbraith S, Mallewa M (2013). Detection of herpes viruses in the cerebrospinal fluid of adults with suspected viral meningitis in Malawi. Infection.

[35] Selim HS, El-Barrawy MA, Rakha ME, Yingst SL, Baskharoun MF (2007). Microbial study of meningitis and encephalitis cases. J Egypt Public Health Assoc.

[36] Gnann JWJ, Whitley RJ (2002). Herpes Zoster. N Engl J Med.

[37] Schoub BD, Johnson S, McAnerney JM (1985). Prevalence of antibodies to varicella zoster virus in healthy adults. South Afr Med J Suid-Afr Tydskr Vir Geneeskd.

[38] Admani B, Macharia WM, Were F. Seroprevalence of varicella zoster antibodies among children with malnutrition, malignancies and HIV infection. East Afr Med J. 2008; 10.4314/eamj.v85i10.9669 19537424

[39] Asiki G, Stockdale L, Kasamba I, Vudriko T, Tumwekwase G, Johnston T (2015). Pilot study of antibodies against varicella zoster virus and human immunodeficiency virus in relation to the risk of developing stroke, nested within a rural cohort in Uganda. Trop Med Int Health TM IH.

[40] Hannachi N, Marzouk M, Harrabi I, Ferjani A, Ksouri Z, Ghannem H (2011). Seroprevalence of rubella virus, varicella zoster virus, cytomegalovirus and parvovirus B19 among pregnant women in the Sousse region, Tunisia. Bull Soc Pathol Exot 1990.

[41] Schaftenaar E, Verjans GMGM, Getu S, McIntyre JA, Struthers HE, Osterhaus ADME, et al. High seroprevalence of human herpesviruses in HIV-infected individuals attending primary healthcare facilities in rural South Africa [internet]. PLoS One. 2014; Available from: http://www.plosone.org/article/fetchObject.action?uri=info%3Adoi%2F10.1371%2Fjournal.pone.0099243&representation=PDF 10.1371/journal.pone.0099243 PMC4051661 24914671

[42] Hannachi N, Marzouk M, Harrabi I, Ferjani A, Ksouri Z, Ghannem H (2011). Seroprevalence of rubella virus, varicella zoster virus, cytomegalovirus and parvovirus B19 among pregnant women in the Sousse region, Tunisia. Bull Société Pathol Exot 1990.

[43] Cost of Vaccinating Refugees Overseas Versus After Arrival in the United States, 2005 [Internet]. [cited 10 Oct 2016]. Available from: https://www.cdc.gov/mmwr/preview/mmwrhtml/mm5709a2.htm 18322445

[44] Ajayi GO, Omilabu SA, Alamu D, Balogun Y, Badaru S (2011). Seroprevalence of other antibodies (herpes, CMV, rubella, varicella, hepatitis B and C, syphilis, chlamydia, mumps, toxoplasmosis) in HIV-positive patients. Clin Exp Obstet Gynecol.

[45] Ghebrekidan H, Rudén U, Cox S, Wahren B, Grandien M (1999). Prevalence of herpes simplex virus types 1 and 2, cytomegalovirus, and varicella-zoster virus infections in Eritrea. J Clin Virol Off Publ Pan Am Soc Clin Virol.

[46] Forbes HJ, Thomas SL, Langan SM (2012). The epidemiology and prevention of herpes zoster. Curr Dermatol Rep.

[47] Lolekha S, Tanthiphabha W, Sornchai P, Kosuwan P, Sutra S, Warachit B (2001). Effect of climatic factors and population density on varicella zoster virus epidemiology within a tropical country. Am J Trop Med Hyg.

[48] de Melker H, Berbers G, Hahné S, Rümke H, van den Hof S, de Wit A (2006). The epidemiology of varicella and herpes zoster in The Netherlands: implications for varicella zoster virus vaccination. Vaccine.

[49] Choo PW, Donahue JG, Manson JE, Platt R (1995). The epidemiology of varicella and its complications. J Infect Dis.

[50] Boëlle PY, Hanslik T (2002). Varicella in non-immune persons: incidence, hospitalization and mortality rates. Epidemiol Infect.

[51] Bardach A, Cafferata ML, Klein K, Cormick G, Gibbons L, Ruvinsky S (2012). Incidence and use of resources for chickenpox and herpes zoster in Latin America and the Caribbean--a systematic review and meta-analysis. Pediatr Infect Dis J.

[52] Jd S, Hoover DR, Shi Q, Mutimura E, Cohen HW, Anastos K (2015). Prevalence of shingles and its association with PTSD among HIV-infected women in Rwanda. BMJ Open.

[53] Blank LJ, Polydefkis MJ, Moore RD, Gebo KA (2012). Herpes zoster among persons living with HIV in the current antiretroviral therapy era. J Acquir Immune Defic Syndr 1999.

[54] Moanna A, Rimland D (2013). Decreasing incidence of herpes zoster in the highly active antiretroviral therapy era. Clin Infect Dis.

[55] dos Reis HLB, Cavalcante FS, dos Santos KRN, Passos MRL (2011). Ferreira D de C. Herpes zoster as a sign of AIDS and nonadherence to antiretroviral therapy: a case report. Clinics.

[56] Negin J, Mills EJ, Bärnighausen T (2012). Aging with HIV in Africa: the challenges of living longer. AIDS Lond Engl.

[57] Meyer PA, Seward JF, Jumaan AO, Wharton M (2000). Varicella mortality: trends before vaccine licensure in the United States, 1970-1994. J Infect Dis.

[58] Adams J, King C, Hook D. Global research report - Africa. Evidence [Internet]. 2010 [cited 19 Aug 2016]; Available from: http://www.eldis.org/document/A51711.

