# Supplementary material for: Varicella zoster virus-associated morbidity and mortality in Africa – a systematic review
Source: BMC Infect Dis. 2017 Nov 14;17:717. doi: 10.1186/s12879-017-2815-9 (PMC5686819; doi:10.1186/s12879-017-2815-9)
Supplement: Supplementary file 2 — Bias Scoring Agreement. Interrater agreement of risk of bias and quality assessment. (DOCX 11 kb) [file 12879_2017_2815_MOESM2_ESM.docx]

**Table S2: Bias scoring on overall (both internal and external) validity**

**Rater 1 versus Rater 2 Crosstabulation**

Counts

|  | **Rater 2** | | | | | Total |
| --- | --- | --- | --- | --- | --- | --- |
|  | **6** | **7** | **8** | **9** | **10** |  |
| **Rater 1** |  |  |  |  |  |  |
| **6** | 1 | 0 | 0 | 0 | 0 | 1 |
| **7** | 0 | 1 | 0 | 0 | 0 | 1 |
| **8** | 0 | 0 | 6 | 4 | 0 | 10 |
| **9** | 0 | 0 | 0 | 3 | 3 | 6 |
| **10** | 0 | 0 | 0 | 0 | 2 | 2 |
|  |  |  |  |  |  |  |
| Total | 1 | 1 | 6 | 7 | 5 | 20 |

| Symmetric measures | Value |
| --- | --- |
| Measure of agreement (Kappa)  No. of valid cases | 0.510  20 |
